# In-plane structural and electronic anisotropy of nanoporous Pt films formed by oblique angle deposition

**DOI:** 10.1038/s41598-024-73301-2

**Published:** 2024-09-24

**Authors:** Daeju Kim, Dong Yeong Kim, Hyunah Kwon, Jaehee Cho

**Affiliations:** 1 School of Semiconductor and Chemical Engineering, Jeonbuk National University, Jeonju, 54896 Republic of Korea; 2 Major of Semiconductor Engineering, Pukyong National University, Busan, 48513 Republic of Korea; 3 Institute for Molecular Systems Engineering and Advanced Materials, Heidelberg University, INF 225, 69120 Heidelberg, Germany; 4 Max Planck Institute for Medical Research, Jahnstrasse 29, 69120 Heidelberg, Germany

**Keywords:** Oblique angle deposition, Pt film, Pt nanostructures, Electrical anisotropy, Porous film, Materials science, Nanoscience and technology

## Abstract

Nanoporous Pt films fabricated by oblique angle deposition hold potential as electrocatalysts in various energy-related fields owing to their high surface area, structural stability, and adequate conductivity. In this study, we investigated the morphology, porosity, and electrical conductivity of nanoporous Pt thin films and systematically studied their interrelationships. Specifically, we revealed an in-plane anisotropy in the electrical conductivity that correlates with the surface morphology of the film. This anisotropy was evident in the resistance measurements along the in-plane lateral and vertical directions, which aligned well with our simple model. The results emphasize the significance of film morphology in determining the film’s electrical properties. This study contributes to the understanding of the physical properties of Pt films fabricated via oblique angle deposition and offers valuable insights for designing nanoporous films for various applications.

**Supplementary Information:**

The online version contains supplementary material available at 10.1038/s41598-024-73301-2.

## Introduction

The high chemical reactivity and structural stability of Pt make it useful as an electrocatalyst in fuel cell technology and various energy and environmental applications^1,2^. Pt thin films are conventionally employed, even though their limited surface areas reduce their catalytic efficiency^3,4^. Pt nanoparticles are widely used to increase the surface areas of Pt-based catalysts, but they require electrically conductive supports^5–7^. The Pt nanoparticles are often dispersed as active sites on carbon supports that provide a path for charge carriers. Despite their conductivity advantages, such supports present significant challenges, including issues of durability and instability in securing the Pt particles^7,8^.

One promising approach for catalyst design involves the use of nanoporous Pt films, which have significantly increased surface areas while maintaining adequate conductivity^4,9,10^. The oblique angle deposition (OAD) technique, widely employed for fabricating sculptured thin films with well-defined nanostructures^11^, can also be applied to create nanoporous films composed of slanted Pt nanorods^12–14^. These films offer enhanced catalytic efficiency because of their increased surface area available for electrochemical reactions^15^, improved selectivity of the catalytic reactions by presenting preferable crystal planes^16^, and robustness owing to their self-supporting nature^17^. In addition, nanoporous Pt films formed by OAD adhere strongly to specific substrates, ensuring long-term durability and stability^12–14^.

The structural features of nanoporous Pt films and the conduction of electrical charges within them must be understood for designing high-efficiency Pt-based catalysts. While numerous studies have focused on the structural and morphological characterizations of Pt films^11–18^, the specific relationships between morphology and electrical charge conduction, particularly anisotropic charge conduction, have not been extensively explored. Understanding this anisotropy is critical not only for optimizing the films for applications where directional charge conduction may be beneficial, but also for ensuring that the anisotropy does not negatively impact applications requiring uniform electrical properties. Thus, a detailed understanding of this behavior is essential for both leveraging and mitigating anisotropy in nanoporous Pt films, broadening their applicability.

Herein, we study the morphology, porosity, and electrical resistance of nanoporous Pt thin films fabricated using a sputter-based OAD technique on Si substrates, focusing on how these properties relate to the incident angle of the flux. The structural features of the films, such as the angles of the slanted nanorods, porosity, and surface void fraction, vary with changes in the deposition angle. Additionally, their behaviors deviate from the theoretical models, indicating a complex relationship between the deposition parameters and nanostructure formation during OAD. Furthermore, based on experimental observations that align well with a simple model, the in-plane anisotropy in the resistance along the lateral and vertical directions is revealed to be closely correlated with the surface morphology of the films. Therefore, our findings contribute to the understanding of Pt OAD films, which will facilitate film designs for various target applications including electrocatalysts.

## Results and discussion

### Morphology of nanostructured pt film

**Fig. 1:**
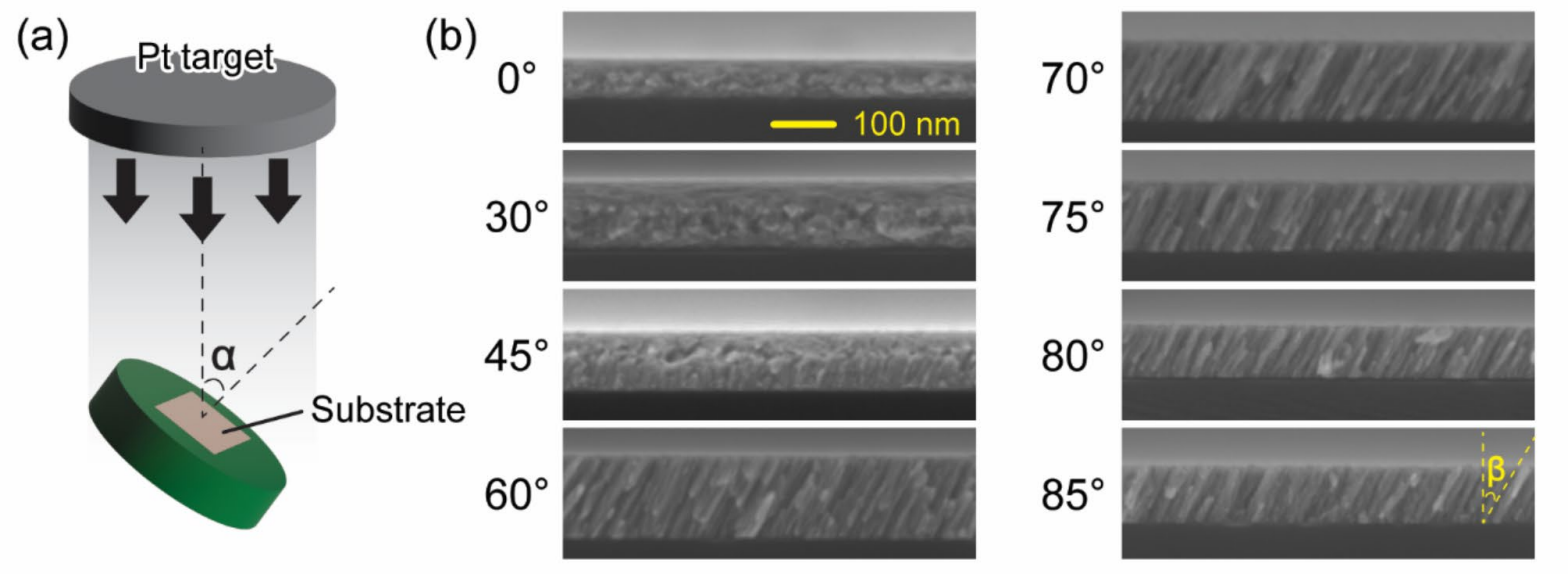
(a) Schematic of the sputter-based OAD process. Here, a Pt target is sputtered, generating a vapor flux directed towards the substrate at an oblique angle α relative to the substrate’s normal. (b) Cross-sectional SEM images of Pt thin films deposited at various incident angles α. The formation of Pt nanocolumns is increasingly evident as the incident angle α increases, especially at and beyond 60°. The nanocolumns are slanted at an angle β relative to the substrate’s normal.

As depicted in Fig. 1a, nanoporous Pt films were deposited using sputtering with the substrate tilted at various oblique angles α relative to the incident flux. The OAD process first forms nuclei that obstruct subsequent vapor flux, thus creating shadowed regions. This shadowing effect results in the growth of slanted rod-like structures. We deposited Pt films at angles α of 0, 30, 45, 60, 70, 75, 80, and 85 degrees, ensuring a consistent nominal deposition rate across all experiments.

Figure 1b presents cross-sectional SEM images illustrating the microstructures of the Pt thin films grown at various angles α. At α = 0°, corresponding to a typical deposition process, dense Pt films are formed. The dense film growth persists up to α = 30°, suggesting that the shadowing effect of the nuclei is not effective at smaller angles. Slanted columnar structures start to emerge at α = 45° and become more prominent at α > 60°. The slanted angle of these columns, designated as β, is measured relative to the substrate’s normal. Additionally, we have performed atomic force microscopy (AFM) on the top surface of the nanoporous Pt thin films, and the results are presented in Supplementary Fig. S1. Surface roughness tends to increase as the substrate tilt angle (α) increases, and slanted columnar structures predominantly emerge at α > 45°.

**Fig. 2:**
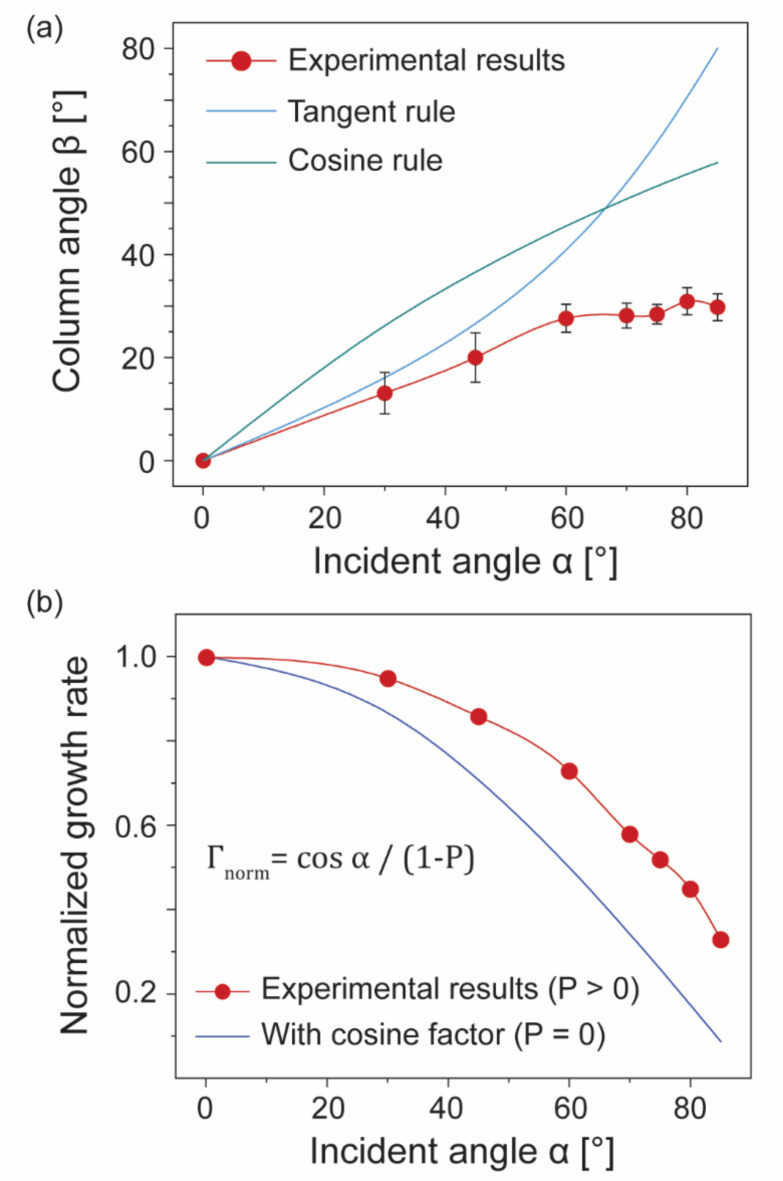
(a) Relationship between the incident angle α and the measured column angle β (represented in red). The average column angle β, as calculated from cross-sectional SEM images of the films, is indicated with error bars. The blue and green lines represent the predicted values of β according to the tangent and cosine rules, respectively. The measured β values exhibit a saturation effect at higher incident angles. (b) Normalized growth rate as a function of α (represented in red), showing a gradual decline. The blue line represents the estimated growth rate based on a cosine factor derived from the area reached by the flux, assuming zero porosity (*P* = 0). The experimental results (red line) demonstrate a higher growth rate due to the positive value of porosity.

Figure 2a displays the relationship between the column angle β and incident angle α. The average column angle β was calculated from ten different positions in cross-sectional SEM images. The angle β shows a clear increase with α for α < 60°, but approaches saturation at larger α values. The correlation between α and β has been empirically established in prior studies, often described using the tangent rule or cosine rule^19,20^. These relationships are defined in Eqs. (1) and (2), which are also illustrated in Fig. 2a.

1 $$\:\text{tan}\alpha\:=2\text{tan}\beta\:$$

2 $$\:{\upbeta\:}=\alpha\:-\text{arcsin}\left(1-\text{cos}\alpha\:\right)/2$$

In this study, the experimentally observed column angle β deviates from both of these established empirical rules. This deviation can be attributed to the scattering of the source flux under relatively high operating pressure conditions. For a working distance of 50 mm, source flux at a temperature of 300 K, and pressures exceeding 1 × 10^− 3^ mbar, the mean free path of the flux is shorter than the working distance. This leads to a deviation in the incident flux from a straight trajectory. Under these conditions, the surface trapping mechanisms are enhanced^11^, and the vapor species interact more intensively with the surface. Such interactions result in increased column diameters and potential column coalescence, thereby producing smaller tilt angles^11^.

The growth rates were calibrated by measuring the height of each porous film with respect to its deposition time. The normalized rates ($\:{\varGamma\:}_{\text{n}\text{o}\text{r}\text{m}}$) are illustrated in Fig. 2b as a function of α. As the source flux remains constant across all depositions, the expected growth rate decreases by a factor of cos α, considering the geometry of the OAD process. However, our measured growth rates exceed the cosine-based estimates, as indicated by the blue line in Fig. 2b. This discrepancy is primarily attributed to the film porosity (*P*), which increases the volume and thickness of the film for a given amount of deposited material. Consequently, the simple cosine rule must be modified to accommodate the effect of the porosity, as shown in the Eq. (3)^21^.

3 $$\:{\varGamma\:}_{\text{n}\text{o}\text{r}\text{m}}=\text{cos}\alpha\:/\left(1-P\right)$$

Then, the porosity is expressed by

4 $$\:P=1-\left(\text{cos}\alpha\:/{\varGamma\:}_{\text{n}\text{o}\text{r}\text{m}}\right)$$

The porosity of the film, estimated using Eq. (4), shows a monotonic increase with the incident angle α, as discussed in detail in section Estimation of surface void fraction and porosity. This demonstrates that sputter-based OAD is advantageous for producing porous nanostructured Pt films with increased surface areas. The UV-Vis measurements are also presented in Supplementary Fig. 2. The reflectance tends to decrease as the deposition angle increases, which can be attributed to the increase in porosity.

### Estimation of surface void fraction and porosity

**Fig. 3:**
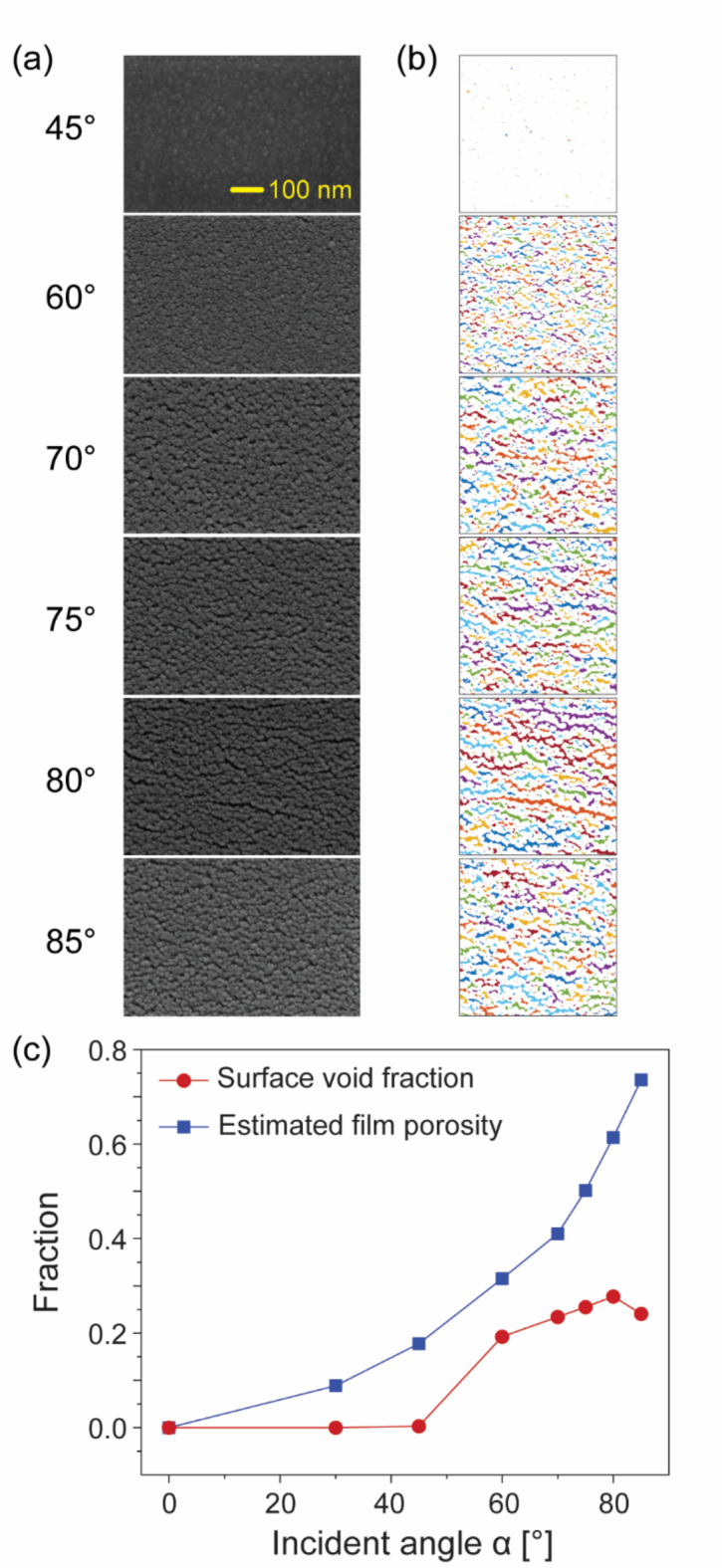
(a) SEM images of Pt thin films, captured along the direction of the Pt columns. (b) Images processed from (a) using adaptive binarization, highlighting the surface void regions in various colors. Different colors are assigned to the separated voids to enhance visibility. (c) Surface void fraction calculated from the binarized images (red) and estimated film porosity (blue) as derived from the theoretical growth rate, plotted as functions of the incident angle α.

The surface void fraction and porosity of the nanostructured Pt film are critical in determining both the Pt surface area and the electronic conductivity of the entire film. To quantify the surface void fraction, we analyzed the tilted top-view SEM images, which were aligned with the nanocolumn angle β, as shown in Fig. 3a. These images were subjected to contrast enhancement to differentiate the Pt nanocolumns from the pores separating them. They were then binarized using a built-in MATLAB function that employed an adaptive method with a sensitivity setting of 0.8. During image processing, the original SEM images were cropped to 900 $\:\times\:\:$900 pixels. The zero elements representing surface voids were colored to enhance visibility, as shown in Fig. 3b.

The surface void fractions were calculated as the ratio of the number of zero elements to the total pixel count (810,000) of each image. The films fabricated at incident angles α < 45° exhibited small surface void areas that were significantly increased at higher α values. The film porosities estimated using Eq. (4) are shown in Fig. 3c. The Pt films exhibited a lower surface void fraction compared to the estimated overall porosity throughout the range of α. This difference is attributed to the broadening of the nanocolumns during OAD^20^. In the competitive growth process, the faster-growing nanocolumns overshadow the others; this effect can be further amplified by a surface-trapping mechanism. Consequently, the surface void fraction at the top of the film is smaller than the initial porosity at the bottom. This broadening effect increases for increased incident angles α, eventually leading to the coalescence of nanocolumn tips and pore closure. The particularly small surface void fraction observed at α = 45° was due to the small initial pore sizes. In our experiments, the surface trapping mechanism, which contributed to the column angle trend discussed in section “Results and discussion”, led to the complete closure of these small pores, resulting in exceptionally small surface void fractions at lower α values.

**Fig. 4:**
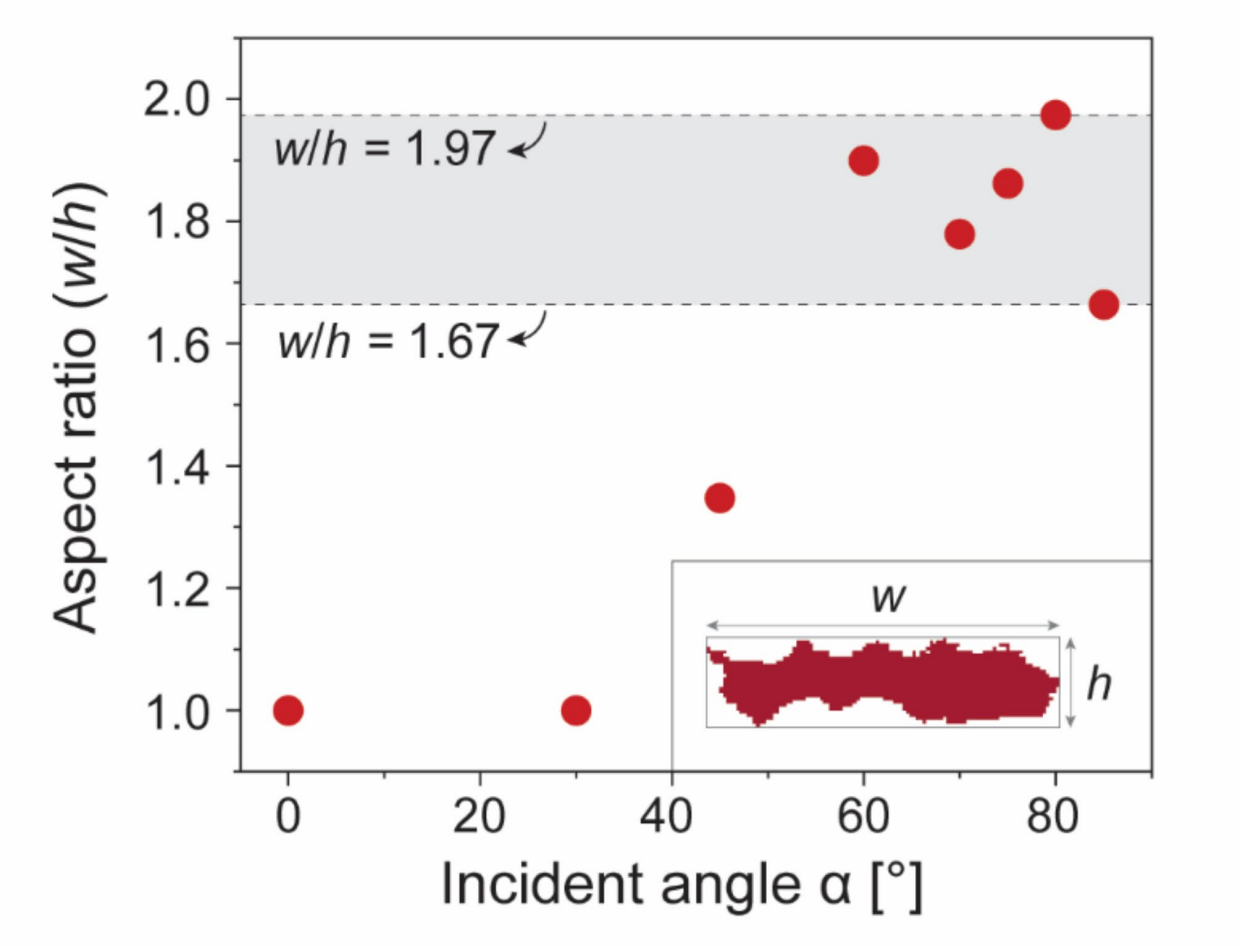
Plot of the average aspect ratios of the surface voids as a function of α, measured as the width (*w*) to height (*h*) ratio. The inset provides a representative example of a surface void, as seen in the processed SEM images. Here, *w* and *h* denote the distances between the end pixels in the lateral and vertical directions, respectively. Notably, the aspect ratios vary from 1.67 to 1.97 at higher incident angles.

The surface voids in the nanostructured Pt films demonstrated anisotropic characteristics, especially at higher incident angles α. A typical surface void derived from the processed SEM images is shown in the inset of Fig. 4. Such voids extend more in the lateral direction (width, *w*) than in the vertical direction (height, *h*). Figure 4 presents the average aspect ratios (*w*/*h*) as a function of α. At incident angles α > 60°, the aspect ratios were significantly higher, ranging from 1.67 to 1.97.

The surface voids in the nanostructured Pt films demonstrated anisotropic characteristics, especially at higher incident angles α. A typical surface void derived from the processed SEM images is shown in the inset of Fig. 4. Such voids extend more in the lateral direction (width, w) than in the vertical direction (height, h). Figure 4 presents the average aspect ratios (w/h) as a function of α. At incident angles α > 60°, the aspect ratios were significantly higher, ranging from 1.67 to 1.97.

This increase in aspect ratios can be attributed to the broadening and eventual coalescence of the tips of the Pt nanocolumns, a phenomenon that was more pronounced in the lateral direction where the shadowing effect was minimized^22,23^. The differential growth rates along the axes due to the shadowing and capture effects resulted in this anisotropic elongation^22^. As the deposition progressed, the capture effect, influenced by varying sticking coefficients, promoted the enlargement and coalescence of the columns, especially in directions orthogonal to the deposition flux^22^. These mechanisms collectively led to the bundling of nanostructures, creating anisotropic domains that are clearly visible when viewed from above^23^. As the nanostructures bundled together, the anisotropy became more pronounced, resulting in the formation of elongated elliptical voids^23^. This bundling effect caused the voids to extend more in the lateral direction (*w*) than in the vertical direction (*h*), significantly contributing to the increased aspect ratios observed at higher incident angles.

This trend of lateral expansion of the voids is not only a structural characteristic but also likely influences the anisotropy of the electrical resistance of the films. The enhanced aspect ratios at higher incident angles suggest that the electrical properties of the films could vary depending on the direction of measurement, potentially impacting their performance in applications where directional conductivity is crucial.

### Electrical properties and their anisotropy

**Fig. 5:**
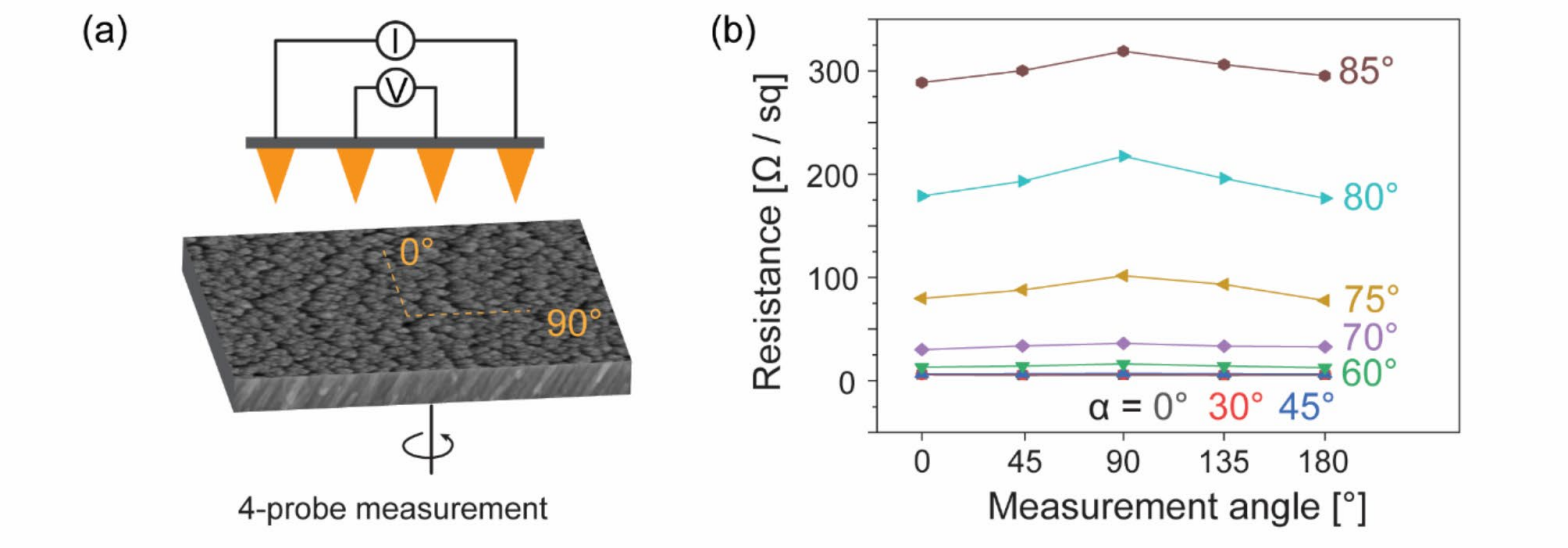
(a) Schematic of the 4-probe measurement setup. The four probes are arranged along a single axis, with the sample undergoing in-plane rotation from 0 to 180°. (b) Sheet resistances of each film measured at various measurement angles.

The sheet resistance values and the anisotropy were evaluated using a 4-probe measurement technique. Although this method is typically used to measure the sheet resistances of homogeneous and compact thin films, we adopted this approach to determine the resistance in a specific direction across the different Pt films^24^. For our measurements, the four probes were aligned along a single axis with an equal spacing of 1.27 mm, as depicted in Fig. 5a. The samples were then rotated in the plane from 0° to 180°. Figure 5b shows the resistance measurements of the various samples as a function of the measurement angle. Supplementary Table 1 presents the average and standard error values for measured resistances, and they are reproducible. The error bars are shown representatively for the sample with an incident angle of 85° in the Supplementary Fig. 3. The resistance values increased with the incident angle α, which can be attributed to the higher porosity at larger α values. More interestingly, the difference in resistance at the measurement angles of 0° and 90° increased with increasing α. The differences in resistance values at measurement angles of 90° and 0° were 1.9, 7.4, 20.7, 33.26, and 50.7 Ω/sq at α = 60, 70, 75, 80, and 85°, respectively. This trend suggests that the morphological anisotropy discussed earlier significantly contributes to the observed anisotropy in the electrical properties.

**Fig. 6:**
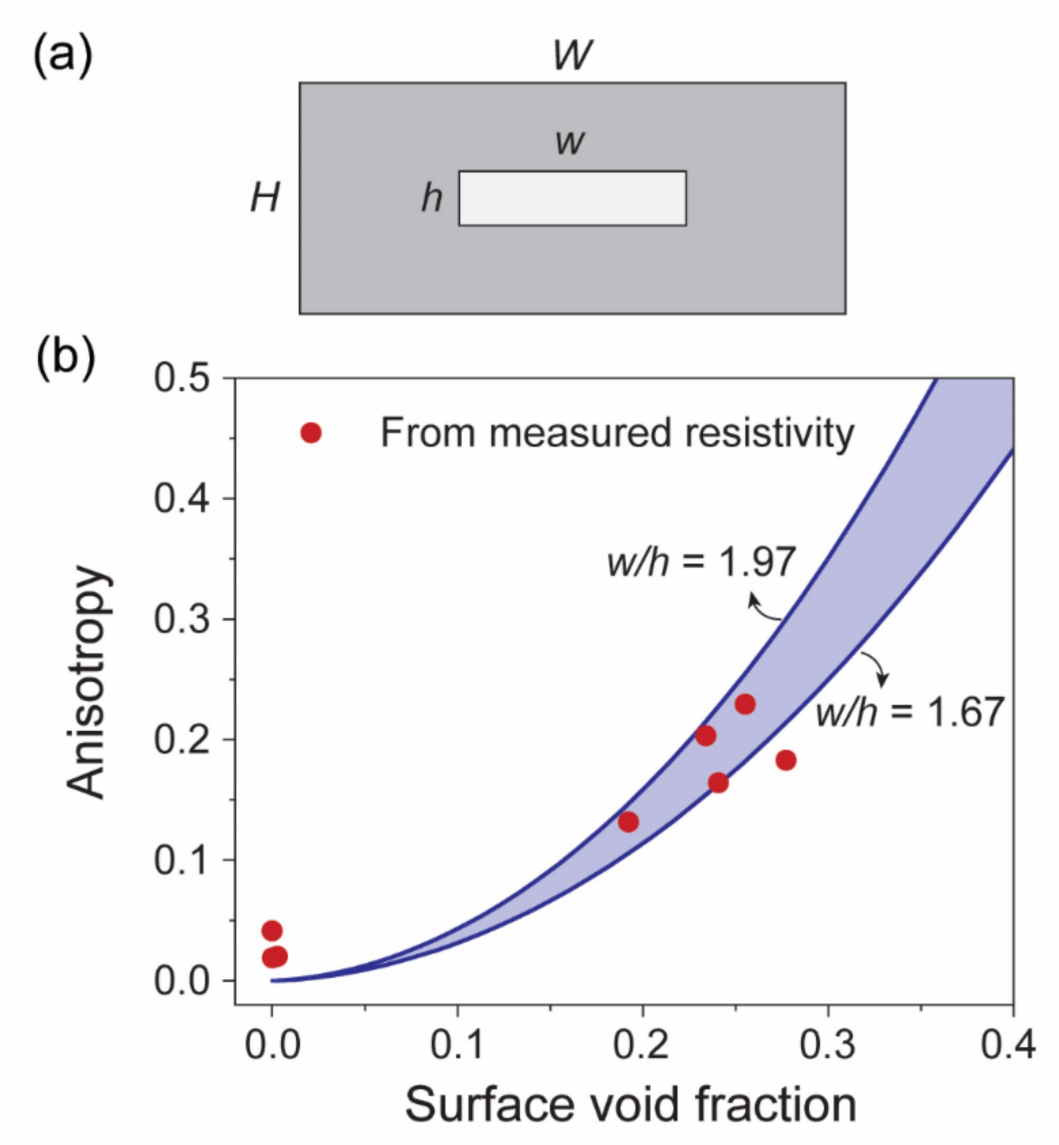
(a) Schematic of a simplified unit comprising interconnected Pt columns (dark gray) and an internal surface void (light gray). *W* and *H* denote the unit width and unit height (considered as 1), while *w* and *h* refer to those of the surface void, respectively. (b) Variation of resistance anisotropy as a function of the surface void fraction. The red symbols depict the experimental resistance anisotropy measured as a function of the surface void fraction, as determined from the SEM images. The blue lines represent the estimated resistance anisotropy calculated from the geometric model shown in (a), assuming surface void aspect ratios in the range of 1.67–1.97. The measured values fall within the range predicted by the model.

To quantify the correlation between the film morphology and the sheet resistance, we visualized the top surface of the Pt film using a simplified model. This model assumes a unit cell composed of connected Pt nanocolumns and a surface void, as shown in Fig. 6a. In this model, *W* and *H* are considered unit lengths denoting the width and height of the unit area, respectively, setting the dimensions of the model to a unit square; *w* and *h* represent the width and height of the surface voids, respectively. Based on this, the resistances in the width and height directions can be expressed using Eqs. (5) and (6), respectively.

5 $$\:{R}_{lateral}\propto\:\frac{W-w}{H}+\frac{w}{H-h}$$

6 $$\:{R}_{vertical}\propto\:\frac{H-h}{W}+\frac{h}{W-w}$$

where $\:{R}_{lateral}$ and $\:{R}_{vertical}$ are the resistances in the width and height directions, respectively. Figure 6b plots the anisotropy of the resistance, which is defined by Eq. (7).

7 $$\:Anisotropy=\frac{\left|{R}_{lateral}-{R}_{vertical}\right|}{{R}_{lateral}}$$

This simplified model yields an estimated resistance anisotropy influenced by the aspect ratio *w/h*. The blue region in Fig. 6b illustrates the estimated resistance anisotropy as a function of the surface void fraction, focusing on the range of 1.67–1.97 from the experimental findings.

The experimental anisotropy was determined in the same manner using the sheet resistance measurements at 0° (lateral direction) and 90° (vertical direction). The results, presented in Fig. 6b as functions of the surface void fraction, closely correspond to those from the simplified model. This strong correlation implies a profound impact of the surface morphology on the sheet resistance. Therefore, the surface trapping mechanism and broadening effect inherent to the OAD process significantly influence the surface morphology and, consequently, the directional charge conduction in the films.

## Conclusions

We explored the structural characteristics of nanoporous Pt films produced via sputter-based OAD and identified anisotropy in the in-plane electrical conduction of these films, which is interconnected with the film nanostructure. Morphological analysis clearly correlates the deposition angle α with the formation of slanted nanocolumns in the Pt films. Pronounced structural anisotropy was observed in the surface voids, particularly at larger values. This anisotropy was quantitatively captured by analyzing the aspect ratios for films with α > 60°, which were noticeably high at 1.67–1.97. The sheet resistance varied by tens of ohms per square depending on the deposition angle α, highlighting the impact of the nanoscale morphology on the electrical properties of the films. Our research provides an in-depth understanding of the intricate interplay among the deposition parameters, morphology, and electrical characteristics of Pt OAD films, offering a foundational framework for the design of nanoporous films using OAD for diverse applications.

## Methods

### Preparation of the Pt films

 A DC magnetron sputtering technique with a Pt target (99.99% purity) was used to deposit Pt on a *p*-type Si (100) substrate. Prior to deposition, the substrate was cleaned using acetone and isopropanol, each with 5 min sonication, rinsed in deionized water for 5 min and dried under N_2_ flow. The substrate was tilted relative to the Pt source flux at angles ranging from 0 to 85°. The working distance of the equipment was set to 50 mm, and the applied current for the deposition was adjusted to 30 mA. The background pressure in the sputtering chamber was maintained at 1.7 × 10^− 3^ mbar prior to Pt sputtering. During Pt sputtering, a mixture of gases was introduced into the chamber with the operating pressure of 8 × 10^− 3^ mbar. Deposition times were extended as the substrate tilt angle (α) increased to account for the decrease in deposition rate. Depositions were performed for 1150, 1200, 1333, 1575, 2000, 2220, 2565, and 3450 s when the substrate was tilted at 0°, 30°, 45°, 60°, 70°, 75°, 80°, and 85°, respectively.

### Microscopy and porosity estimation

 The microstructures of the films were characterized by scanning electron microscopy (SEM) using an Apreo S apparatus (Thermo Fisher Scientific) operated at 5 kV. For the cross-sectional images, the samples were cut in the direction of the flux to observe the angles of the nanocolumns. Tilted top-view images were captured at angles corresponding to the nanocolumn angles to maximize the contrast between the Pt nanocolumns and pores, thus allowing a clear estimation of the surface void fraction. The SEM images were converted into binary images using a built-in MATLAB function operated using an adaptive method with a sensitivity setting of 0.8. The analyzed images had a resolution of 900 × 900 pixels. The surface void fraction was calculated as the ratio of the number of “0” elements (pores) to “1” elements (solids) in the binarized image.

### Four-probe measurement

 A commercial 4-probe measurement system (CMTSR1000N) was used to characterize the sheet resistances of the Pt OAD films. The system consisted of four probes aligned along one axis with an equal spacing of 1.27 mm. While 4-probe measurements are generally used for homogeneous and dense films, this one-dimensional equal-spacing configuration is also appropriate for characterizing the directional dependence of charge conduction properties in Pt films. The measurements were conducted by rotating the sample in-plane by 45° each time to analyze the anisotropy of the resistance parallel and perpendicular to the source flux direction.

## Electronic supplementary material

Below is the link to the electronic supplementary material.

Supplementary Material 1

## Data Availability

The datasets used and analysed during the current study available from the corresponding author on reasonable request.
