# Supplementary material for: In-plane structural and electronic anisotropy of nanoporous Pt films formed by oblique angle deposition
Source: Sci Rep. 2024 Sep 24;14:22013. doi: 10.1038/s41598-024-73301-2 (PMC11422490; doi:10.1038/s41598-024-73301-2)
Supplement: Supplementary file 1 — Supplementary Material 1 [file 41598_2024_73301_MOESM1_ESM.docx]

**Supplementary Information**

In-plane structural and electronic anisotropy of nanoporous Pt films formed by oblique angle deposition

Daeju Kim^a^, Dong Yeong Kim^b^, Hyunah Kwon^c^*, and Jaehee Cho^a^*

**Table S1**. Average and standard error values for measured resistances.

| Measurement angle | Average | Standard error | Average | Standard error | Average | Standard error |
| --- | --- | --- | --- | --- | --- | --- |
| Incident angle | α = 0˚ | | α = 30˚ | | α = 45˚ | |
| 0˚ | 5.9127 | 0.184556 | 6.1722 | 0.021356 | 6.6125 | 0.055255 |
| 45˚ | 5.3622 | 0.124556 | 6.1976 | 0.015943 | 6.773 | 0.065863 |
| 90˚ | 5.5925 | 0.17891 | 6.3455 | 0.033093 | 7.118 | 0.063657 |
| 135˚ | 5.3609 | 0.118917 | 6.1502 | 0.030234 | 6.9319 | 0.052164 |
| 180˚ | 5.777 | 0.222423 | 6.2586 | 0.008215 | 6.579 | 0.074744 |
| Incident angle | α = 60˚ | | α = 70˚ | | α = 75˚ | |
| 0˚ | 13.159 | 0.058437 | 30.11403 | 0.34301 | 79.6892 | 0.987406 |
| 45˚ | 14.275 | 0.089065 | 33.66083 | 0.342232 | 87.96772 | 0.977103 |
| 90˚ | 16.326 | 0.089579 | 36.35734 | 0.305786 | 101.7678 | 0.99105 |
| 135˚ | 14.396 | 0.201917 | 33.44043 | 0.26989 | 93.24773 | 0.544984 |
| 180˚ | 12.8 | 0.108185 | 32.85723 | 0.238725 | 77.66419 | 1.903613 |
| Incident angle | α = 80˚ | | α = 85˚ | |  | |
| 0˚ | 178.7696 | 2.857094 | 288.561 | 1.973801 |  |  |
| 45˚ | 193.1636 | 1.474416 | 300.0858 | 1.574587 |  |  |
| 90˚ | 217.2716 | 1.629917 | 319.0723 | 1.061744 |  |  |
| 135˚ | 195.7316 | 2.121907 | 306.0333 | 1.876573 |  |  |
| 180˚ | 176.6336 | 2.27352 | 295.2498 | 2.581526 |  |  |


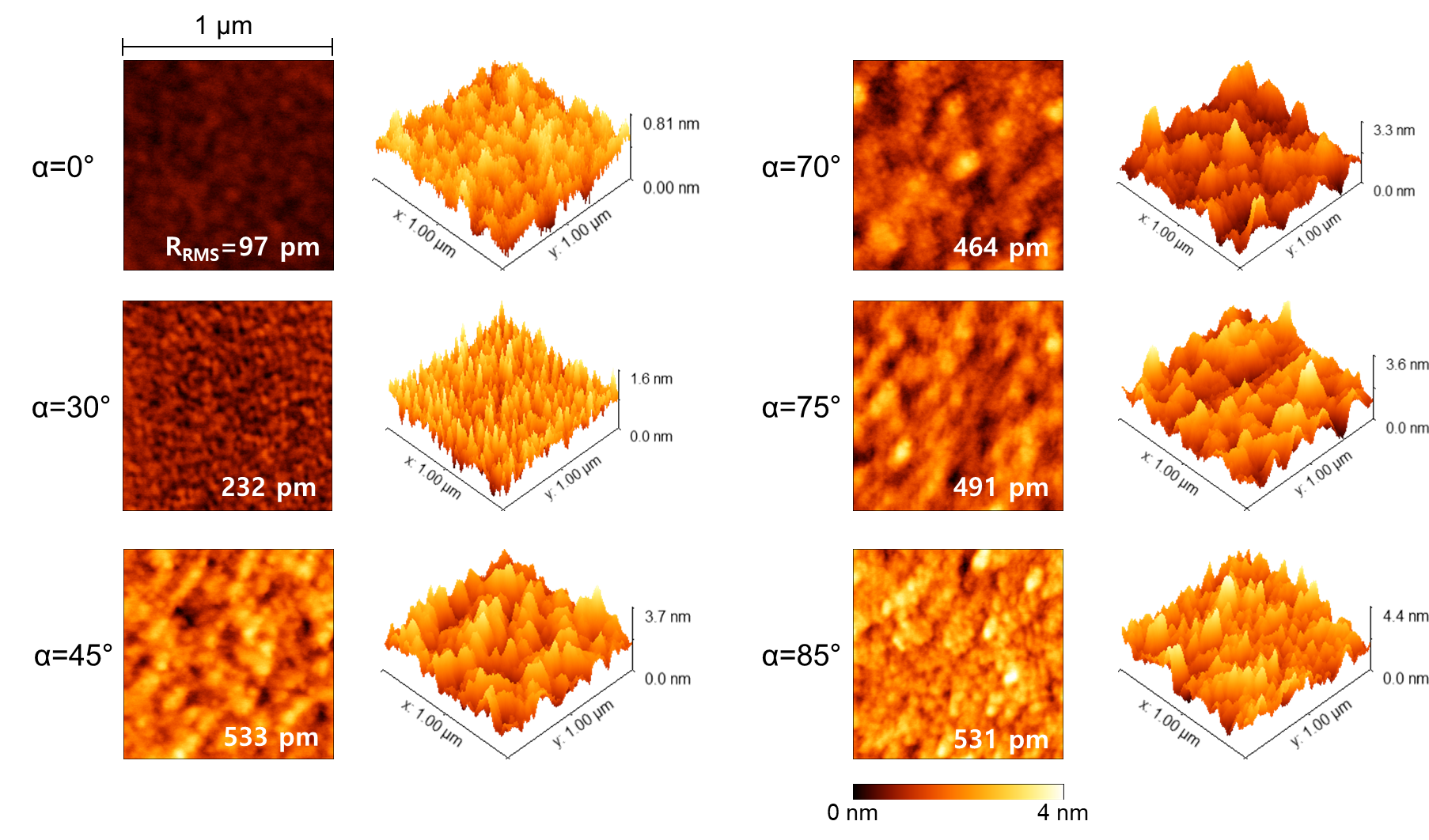


**Figure S1.** Surface morphology and three-dimensional profiles of Pt nanorods deposited at various angles (α), measured by atomic force microscopy. The scanning area is 1 μm × 1 μm, and the height range is 0 to 4 nm for all cases. Root mean square roughness (R_rms_) values are provided in the images.


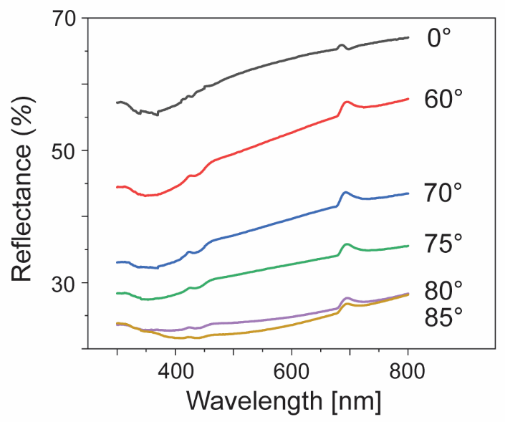


**Figure S2.** Reflectance of Pt nanorods deposited at different angles, ranging from 0° to 85°.


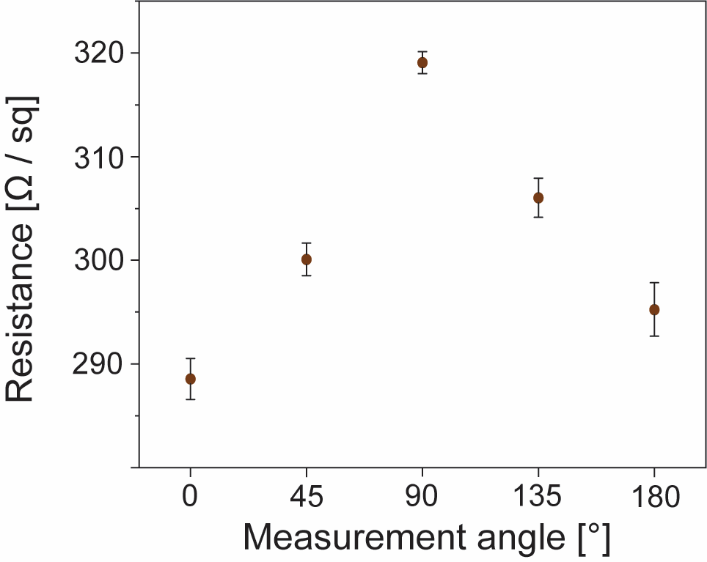


**Figure S3**. Resistances of the sample (α = 85˚) at various measurement angles. Error bars are shown for each data point.
